# Nucleostemin and ASPP2 expression is correlated with pituitary adenoma proliferation

**DOI:** 10.3892/ol.2013.1562

**Published:** 2013-09-04

**Authors:** LIN MA, ZHI-MIN CHEN, XUE-YUAN LI, XIN-JUN WANG, JI-XIN SHOU, XU-DONG FU

**Affiliations:** 1 Department of Neurosurgery, The Fifth Affiliated Hospital of Zhengzhou University, Zhengzhou, Henan 450052, P.R. China; 2 Department of Obstetrics and Gynecology, The First Affiliated Hospital of Zhengzhou University, Zhengzhou, Henan 450052, P.R. China

**Keywords:** apoptosis stimulating of p53 protein 2, Ki-67, nucleostemin, pituitary adenoma, semi-quantitative PCR, sequencing

## Abstract

Nucleostemin is a GTP-conjugated protein located in the nucleoli of stem cells and certain cancer cells, and maintains cellular self-renewal. The present study aimed to evaluate nucleostemin as a potential target for pituitary adenoma gene therapy by investigating nucleostemin and apoptosis-stimulating of p53 protein 2 (ASPP2) expression and their effect on pituitary adenoma cell proliferation. A total of 71 samples of pituitary adenomas were collected. Semi-quantitative PCR was used to detect the expression of nucleostemin and ASPP2 mRNA in the samples. Immunochemistry techniques were used to examine Ki-67 expression in the paraffin section of the samples. Coherent clinical data were also collected. Nucleostemin and ASPP2 were detectable in all the pituitary adenoma samples. Significant differences were observed in nucleostemin and ASPP2 expression between invasive pituitary adenoma and non-invasive pituitary adenomas (P<0.01) and the Ki-67 labeling index (LI; P>0.05). The difference in the Ki-67 LI between the recurrence and non-recurrence groups was significant (P<0.05). There was positive correlation between nucleostemin gene expression and the Ki-67 LI levels (P<0.05). The correlation between ASPP2 expression and the Ki-67 LI was negative (P<0.05). Negative correlation was demonstrated between nucleostemin and ASPP2 expression (P<0.01). The nucleostemin and ASPP2 genes were expressed in the human pituitary adenoma tissues. The differences in the expression of nucleostemin, ASPP2 and Ki-67 in the various pathological types of pituitary adenomas represented differences in molecular biological character and were associated with invasion. In the pituitary adenomas, the expression of nucleostemin and ASPP2 was correlated with tumor proliferation. Nucleostemin, ASPP2 and Ki-67 may serve as valid clinical detection markers for the invasion of pituitary adenomas.

## Introduction

Pituitary adenomas are common intracranial tumors with a rising incidence in China (1). However, the mechanism of occurrence remains unclear. The alteration of p53 gene function has been shown to be closely associated with tumorigenesis, tumor progression and prognosis. p53 gene mutation plays a significant role in malignant tumors, although the incidence of p53 gene mutation is low in pituitary adenomas. Therefore, a functional alteration in the wild-type p53 gene may play a more significant role in pituitary adenomas. Nucleostemin (2) and apoptosis-stimulating of p53 protein 2 (ASPP2) (3,4) are two newly-identified genes that have been shown to correlate with the regulation of p53 gene function. However, in pituitary adenoma, the expression of the nucleostemin and ASPP2 genes and their role in tumor cell proliferation remains unknown. The present study aimed to investigate the expression of nucleostemin, ASPP2 and Ki-67 in pituitary adenomas and lay a foundation for the further research of pituitary tumor occurrence and progression.

## Materials and methods

### Tissue samples

A total of 71 cases of pituitary adenoma resection specimens were collected between January 2004 and August 2005. Associated clinical data were also collected for the present study. The tissues were collected anonymously and this procedure was exempted from the consent requirement. The age range of the patients was 16–72 years (mean, 42.54 years). Among the total cases, 33 were male was and 38 were female at a ratio of 0.87:1. The shortest course of the disease was 15 days and the longest was 18 years (mean, 39.58 months). The tumor diameter ranged between 0.8 and 7 cm (mean diameter, 2.61 cm). Among the total cases, eight were micro adenomas (11.27%), 50 were large adenomas (70.42%) and 13 were huge adenomas (18.31%). Of the 71 patients, 64 cases were followed up and seven were lost. The rate of follow-up was 90.1%. The range of follow-up duration was 8–26 months. Five cases of recurrence were observed during the follow-up period.

The invasive characteristics of the tumor were judged according to the Wilson modified Hardy classification (5). Among the total 71 cases, invasive pituitary adenomas were identified in 36 cases (50.70%) and non-invasive pituitary adenomas in 35 cases (49.30%).

### Semi-quantitative PCR

The nucleostemin and ASPP2 mRNA expression levels were determined using semi-quantitative PCR. The PCR primer was designed according to the reported gene sequence of nucleostemin, ASPP2 mRNA and β-actin in GenBank (Table I). Due to the of low amplification of ASPP2, nest PCR (N-PCR) was used in order to enhance the sensitivity and specificity of detection.

#### Methods of qPCR

i) Total RNA extraction. The total RNA was extracted following cell lysis using TRIzol, and the RNA quality was determined; ii) cDNA compounding. The volume of the reverse transcription reaction system was 30 μl, and the reaction conditions were 42°C for 60 min and 95°C for 5 min. The final cDNA product was conserved at 4°C; and iii) PCR. The compounded cDNA was used as a template for PCR. Gene fragments of nucleostemin and ASPP2 were achieved, separately. The PCR amplification product (6 μl) was used to perform 2% agarose gel electrophoresis and the optical density (OD) was calculated for nucleostemin, ASPP2 and β-actin. Finally, a ratio between the three was obtained.

#### Sequencing

**Figure 1:**
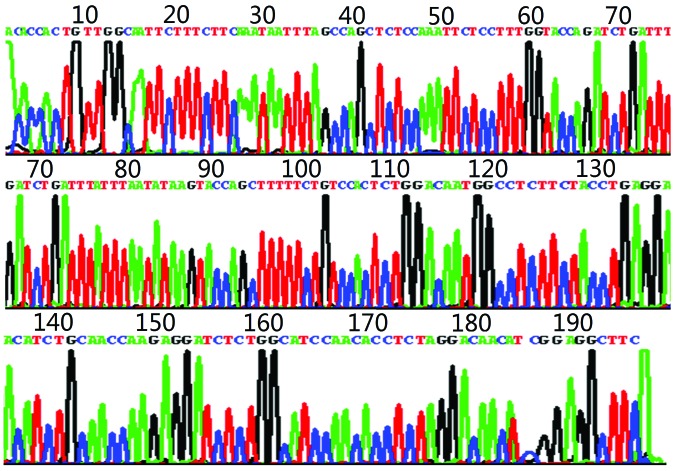
PCR amplification fragment fully corresponding to the target gene, nucleostemin.

**Figure 2:**
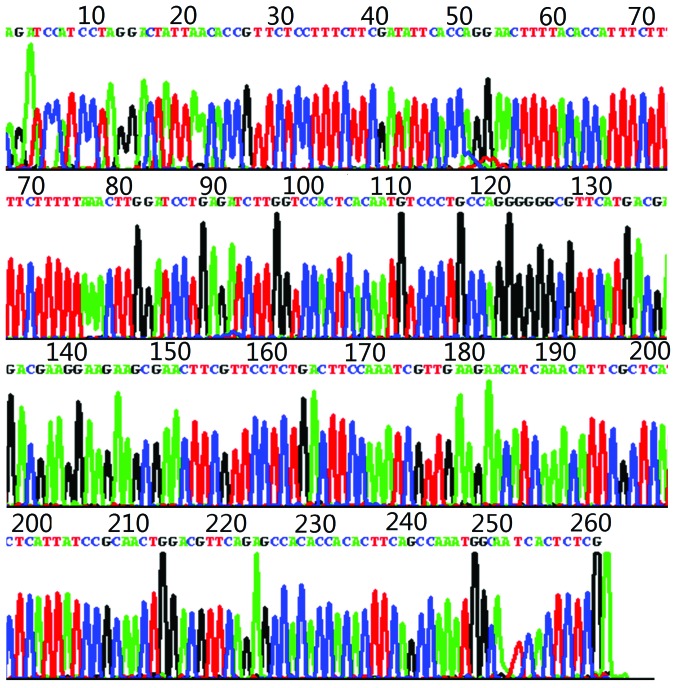
PCR amplification fragment fully corresponding to the target gene, apoptosis-stimulating of p53 protein 2 (ASPP2).

Subsequent to separating and purifying the amplification banding, sequencing was performed to verify the sequence. The aforementioned experiment was performed by Shanghai Sheng-Gong Biological Engineering Technology Service Co., Ltd. (Shanghai, China) and analyzed using Blast software (http://blast.ncbi.nlm.nih.gov/Blast.cgi). The sequence of the amplification fragment was confirmed to correspond to the target gene completely (Figs. 1 and 2).

### Immunohistochemistry for detecting Ki-67 expression

MIB-1 monoclonal antibody immunohistochemical staining was used to detect Ki-67 expression. The primary antibody was rat anti-human Ki-67 monoclonal antibody (1:100), the secondary antibody was goat anti-rat IgG (1:100) and the tertiary antibody was horseradish peroxidase marker chain mildew avidin. The reagents were all obtained from Beijing Zhongshan Biological Technology Co., Ltd., (Beijing, China).

The results were defined as positive or negative according to whether Ki-67 staining was observed in the nucleus. On each slide, five high-power fields were selected randomly. The numbers of total and positive cells were counted and the percentage of positive cells from the total cells was calculated as the Ki-67 mark index. The calculation formula was as follows: Number of positive cells/total cells × 100.

### Statistical analysis

The results are presented as the means ± standard deviation. The experimental data were analyzed and processed using SPSS 11.5 statistical software (SPSS, Inc., Chicago, IL, USA). P<0.05 was considered to indicate a statistically significant difference.

## Results

### ASPP2 mRNA and Ki-67 expression in human pituitary adenomas

**Figure 3:**
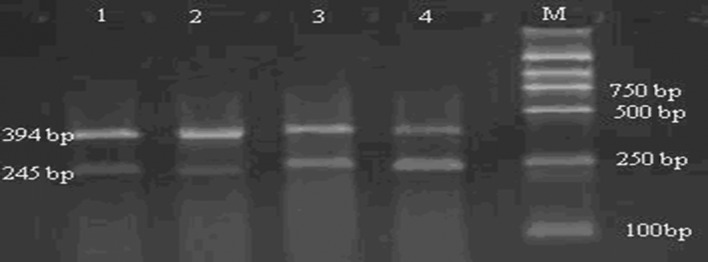
Agarose gel electrophoresis of nucleostemin (245 bp) and β-actin (394 bp) mRNA in pituitary adenomas.

**Figure 4:**
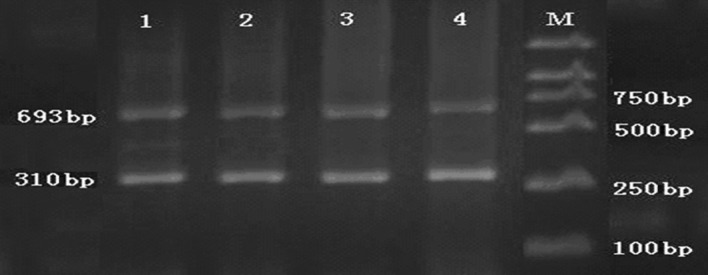
Agarose gel electrophoresis of apoptosis-stimulating of p53 protein 2 (ASPP2; 310 bp) and β-actin (693 bp) mRNA in pituitary adenomas.

**Figure 5:**
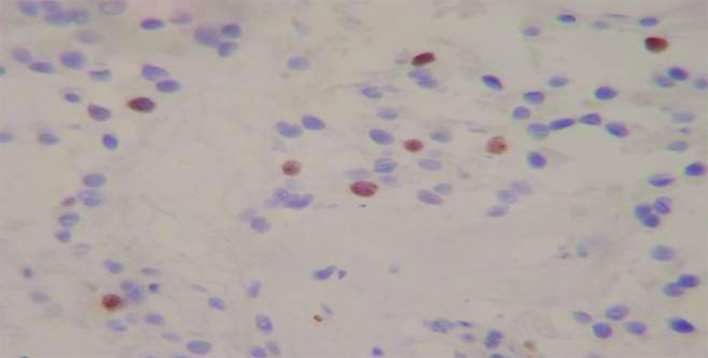
Immunohistochemical staining of Ki-67 expression in pituitary adenomas (magnification, ×40).

Nucleostemin, ASPP2 mRNA and Ki-67 expression was observed in all the 71 cases of pituitary adenomas (Figs. 3–5). The statistical results revealed that a positive correlation existed between the nucleostemin mRNA levels and Ki-67 (r=0.237; P<0.05). A negative correlation was observed between the ASPP2 mRNA expression levels and Ki-67 (r=−0.256; P<0.05), and a negative correlation was observed between nucleostemin and ASPP2 mRNA levels (r=−0.340; P<0.01).

### Comparison of the indicators in various pathological pituitary adenomas

The pathological pattern was classified according to the immunohistochemical staining of the pituitary hormone. In all 71 cases, there were 14 growth hormone (GH), 13 prolactin (PRL), five adrenocorticotropic hormone (ACTH), seven gonadotropic, 10 non-functional and 22 plurihormonal adenomas.

The expression of nucleostemin, ASPP2 mRNA and Ki-67 in the various pathological patterns are shown in Table II. The statistics demonstrated no significant difference between nucleostemin and ASPP2 mRNA expression in the various pathological patterns (P>0.05). However, a significant difference existed between the Ki-67 LI (P<0.05). The expression level of Ki-67 was highest in the PRL adenomas (Ki-67 LI, 3.63±0.84) and lowest in the non-functional adenomas (Ki-67 LI, 2.18±1.08). When compared with each other, the diversity of Ki-67 LI in the PRL and non-functional adenomas was varied (P<0.01). The Ki-67 LI level between the PRL and gonadotropic adenomas (P<0.05), the non-functional and GH adenomas (P<0.05) and the non-functional and plurihormonal adenomas (P<0.05) differed from each other significantly.

### Comparison of various indicators in invasive and non-invasive pituitary adenomas

Nucleostemin expression levels and the Ki-67 LI were higher in the invasive adenomas compared with the non-invasive adenomas. However, for ASPP2, the invasive adenomas exhibited lower levels (Table III). The difference in nucleostemin and ASPP2 expression between the invasive and non-invasive adenomas was of notable significance (P<0.01) and the difference in the Ki-67 LI was also significant (P<0.05).

### Comparison of various indicators in recurrent and non-recurrent pituitary adenomas

Among the 64 cases that were followed up, five experienced recurrence (7.8%). In the recurrence group, the expression of nucleostemin and Ki-67 LI was higher compared with the non-recurrence group. However, the expression of ASPP2 was lower compared with the non-recurrence group (Table IV). The statistical results revealed that for the Ki-67 LI, significant differences existed between the two groups (P<0.05). However, the nucleostemin and ASPP2 expression levels were not significantly different (P>0.05).

## Discussion

Pituitary adenomas are common intracranial tumors that account for ~10% of all intracranial tumors. According to their biological behavior, pituitary adenomas may be divided into invasive and non-invasive subgroups. Although the pathogenesis of pituitary adenomas is a multi-step process containing multiple factors, the exact pathogenesis remains unclear.

Nucleostemin is a relatively new p53 binding protein that was first reported by Tsai and McKay (2) in 2002. Nucleostemin is located in the nucleus of stem and tumor cells and is involved in the regulation of stem and tumor cell proliferation. The protein maintains the proliferation state of stem cells and inhibits their differentiation into mature cells. Although the expression of nucleostemin was highly detected in human tumor cells, the protein does not exist in terminally differentiated cells.

Previous studies have shown that nucleostemin combines and reacts with p53 in the nucleoplasm. Based on existing experimental evidence, a pattern of action for nucleostemin may be proposed. Nucleostemin exists in the nucleolus prior to binding with GTP and is then transferred back to the nucleolus afterwards, where it recombines with p53 protein and inhibits its growth inhibition function. Cell differentiation leads to a decline in nucleostemin gene expression, and p53 is released and activated. Finally, activated p53 is able to induce certain genes to be expressed, which allow cells to exit the mitotic cycle and differentiate (2,6).

Tsai and McKay (7) also reported a dynamic mechanism of nucleostemin moving across the nucleolus and nucleoplasm. Nucleostemin regulates its combined state with GTP using its amino terminal, thus adjusting the amount of nucleostemin protein in the nucleolus and nucleoplasm. Under the effects of internal and external signals, cells may bi-directionally and rapidly regulate the amount of nucleostemin protein in the nucleolus. Therefore, nucleostemin combines with p53 to form a complex, affecting the function of cell cycle regulation and cell apoptosis induction. The increased expression of nucleostemin may enhance the percentage of S-phase cells and increase the rate of tumor growth. When siRNA was used to inhibit nucleostemin expression in the HeLa cell line, the percentage of S-phase cells decreased, the percentage of G_0_/G_1_ stage cells increased, cell proliferation reduced significantly and cell tumorigenicity decreased (8).

The ASPPs are a recently discovered protein family containing three members, ASPP1, ASPP2 and iASPP (9). Subsequent to forming complexes with p53, ASPPs produce various effects on cell apoptosis. ASPP1 and ASPP2 may spur the combination of the apoptosis gene promoter of p53 with DNA, thus heightening the cell apoptosis function of p53 (3,4). iASPP combines with the p53 binding site, competing with ASPP1 and ASPP2, thus reducing the tumor suppression function of p53 (9).

Numerous studies have investigated how the ASPP family regulates p53 function. Llanos *et al*(10) formed the co-expression of ASPP1 or ASPP2 with p53 through transfection and observed that apoptosis occurred in 50% of the transfected tumor cell lines. The co-expression of ASPP1 or ASPP2 with tumor suppressor genes, such as E2Fi and Bax, resulted in no change in the amount of apoptotic cells. In addition, ASPP2 expression was observed to be associated with clinical prognosis (11). A study by Lossos *et al*(11) on diffused large B-cell lymphoma and follicular lymphoma revealed that the survival time of patients with higher levels of ASPP2 was longer than those with lower levels of ASPP2. Zhu *et al*(12) revealed that the ASPP2 protein level and apoptotic function were regulated by mechanisms of proteasome degradation, which participated in the p53-ASPP2 apoptotic pathway. In contrast, Mdm and mdmX were shown to block the p53 apoptotic effect that was induced by ASPP1 and ASPP2 (13). A study suggested that ASPP is a crucial regulatory protein for p53 *in vivo* and that ASPP1 and ASPP2 may enhance p53-dependent apoptosis through increasing the vitality of the original apoptosis gene (14).

Ki-67 is a nuclear protein that is associated with cell proliferation. The protein is regarded as a good indicator to evaluate cell proliferation as it is expressed in each period of cell proliferation. The ratio of Ki-67-positive cells in all cells (Ki-67 LI) is often used to evaluate cell proliferation. Previous studies have shown that the Ki-67 LI is associated with the invasive ability of pituitary adenomas (15–19). One study revealed that the levels of Ki-67 LI in various pathological patterns of pituitary adenomas differed, indicating that the proliferative activity of the various endocrine types also differed (20). Another study identified that the Ki-67 LI was significantly higher in recurrent pituitary adenomas than in primary tumors, which indicated that the Ki-67 LI may be used to judge recurrence and prognosis (21).

The present study identified that the expression of nucleostemin and ASPP2 may be detected in pituitary adenomas. However, ASPP2 expression was significantly higher in the non-invasive pituitary adenomas than in the invasive adenomas. Nucleostemin expression was significantly higher in the invasive pituitary adenomas than in the non-invasive adenomas. In the pituitary adenomas, nucleostemin expression was negatively correlated with ASPP2 expression. ASPP2, but not nucleostemin gene expression, was positively correlated with the Ki-67 LI. Higher nucleostemin gene expression, lower ASPP2 gene expression and higher Ki-67 expression correlated with each other, indicating that changes in all three factors occurred simultaneously or concomitantly in pituitary adenoma tumorigenesis and biological alterations, and that there may be a causal connection. The three factors may regulate the cell proliferative status through a specific mechanism. An increase in nucleostemin expression and a reduction in ASPP2 caused the inhibition of the cell apoptotic function of p53. Therefore, the tumor cell proliferation ability was strengthened and the invasive character improved.

In conclusion, nucleostemin and ASPP2 were expressed in pituitary adenoma tissues and were associated with the aggressive behavior and proliferation activity of the tumor, indicating that these factors play a significant role in pituitary adenoma oncogenesis and tumor progression, and have the potential to be a target for pituitary adenoma gene therapy. However, the way the factors are combined to regulate the proliferation state requires further study.

## Figures and Tables

**Table I:** Primer sequence and amplification fragment length of nucleostemin and ASPP2 for PCR.

Gene	Primer sequence	Product length, bp	GeneBank no.
Nucleostemin			
Upstream	GAAGCCTCCGATGTTGTCCT	245	NM014366
Downstream	CACACGCTTGGTTATCTTCCC		
β-actin (nucleostemin)			
Upstream	CCCAGAGCAAGAGAGGCATCC	394	NM001101
Downstream	AGGTAGTCAGTCAGGTCCCG		
ASPP2			
R1	TGCCGATGTTTCTTACCGTG	310	NM001031685
R2	CGAGAGTGATTGCCATTTGG		
R3	AATCTGTTGCTGCTGGCGAG		
R4	ACTTGTTGCTGTTGTCGCTG		
β-actin (ASPP2)			
β1	GAGACCTTCAACACCCCAGC	694	NM001101
β2	CCCAGAGCAAGAGAGGCATCC		
β3	ACATCTGCTGGAAGGTGGAC		

ASPP2, apoptosis-stimulating of p53 protein 2.

**Table II:** Expression of nucleostemin, ASPP2 mRNA and Ki-67 in various pathological pituitary adenomas.

Pathological type	Nucleostemin	ASPP2	Ki-67 LI, %
GH adenomas (n=14)	0.47±0.41	1.12±0.64	3.13±1.12
PRL adenomas (n=13)	0.56±0.16	0.46±0.22	3.63±0.84
ACTH adenomas (n=5)	0.39±0.23	0.81±0.47	2.82±0.85
Gonadotropic adenomas (n=7)	0.31±0.47	0.84±0.74	2.48±0.81
Non-functional adenomas (n=10)	0.21±1.81	0.73±0.49	2.18±1.08
Plurihormonal adenomas (n=22)	0.45±0.53	0.69±0.64	2.99±1.08
Total (n=71)	0.42±0.40	0.76±0.58	2.96±1.07

Data presented as the means ± SD. ASPP2, apoptosis-stimulating of p53 protein 2; GH, growth hormone; PRL, prolactin; ACTH, adrenocorticotropic hormone.

**Table III:** Expression of nucleostemin, ASPP2 mRNA and Ki-67 in invasive and non-invasive pituitary adenomas.

Group (n=71)	Nucleostemin	ASPP2	Ki-67 LI, %
Invasive adenomas (n=35)	0.55±0.49^a^	0.55±0.46^a^	3.28±1.09^b^
Non-invasive adenomas (n=36)	0.30±0.22	0.97±0.62	2.65±0.97

a P<0.01 vs. non-invasive adenomas;

b P<0.05 vs. non-invasive adenomas.

Data are presented as the means ± SD. ASPP2, apoptosis-stimulating of p53 protein 2.

**Table IV:** Expression of nucleostemin, ASPP2 mRNA and Ki-67 in recurrent and non-recurrent pituitary adenomas.

Recurrent	Nucleostemin	ASPP2	Ki-67 LI, %
Yes (n=5)	0.56±0.23	0.39±0.17	4.11±1.39^a^
No (n=59)	0.40±0.31	0.77±0.59	2.87±1.04

a P<0.05 recurrent vs. non-recurrent pituitary adenomas.

Data are presented as the means ± SD. ASPP2, apoptosis-stimulating of p53 protein 2.
